# Close of Vol. III

**Published:** 1888-06

**Authors:** 


### CLOSE OF VOLUME THREE.

This issue completes VOLUME THREE of THE JOURNAL. We return hearty thanks to the numerous friends and patrons of THE JOURNAL who have so generously seconded our efforts to give to the profession of Texas a periodical worthy of them and of the great State. THE JOURNAL is a success; and at the close of this, the third year, the outlook is still bright and full of promise, and we begin the *fourth round* with the approval of our own conscience and that of many friends; with an augmented circulation, and a flattering advertising patronage, and so far all things are lovely.

Every effort will be made to still further improve THE JOURNAL and to make it indispensable to every member of every organized body in the State, especially; and a valuable aid to the general practitioner.

There are some who, doubtless, in the press of business, have forgotten the little matter of subscription (which is payable *in advance*) and have let it run on, now, a year, (in a few instances, two and even three years—but we could count the latter on our fingers). We call on them now, to show their appreciation of the long indulgence, by a prompt settlement of arrears, and a prompt renewal.

We take occasion to say here, that the publication day will be, as heretofore, about the 25th of each month. Some so love the favorite that if it is not at hand by the 20th to 25th, they begin to write "to know the reason why." Be patient. We address the wrappers and mail THE JOURNAL to every one of you, with our own hands, and none are overlooked. The May number was late on account of the week's festivities, (Drill and Dedication,) which everybody, printers and all, indulged in, except yours truly; and this number is gotten out a few days earlier, so as to give time to the publication of the Transactions of the Texas State Medical Association.

Vol. IV., No. 1, July, 1888, will make its appearance (D. V.) about the 25th of the month, with its best bow, and best wishes to its numerous friends.

